# Supplementary material for: Functional brain connectivity in early adolescence after hypothermia-treated neonatal hypoxic-ischemic encephalopathy
Source: Pediatr Res. 2025 Mar 2;98(5):1827–34. doi: 10.1038/s41390-025-03951-z (PMC12602361; doi:10.1038/s41390-025-03951-z)

Supplemental Table 2 Clinical and cognitive outcome stratified by exposure to HT-treated neonatal HIE. Abbreviations: WISC-V, Wechsler Intelligence Scale for Children fifth version; IQR, Interquartile Range; FSIQ, Full Scale Intelligence Quote; CP, Cerebral Palsy; ADHD, Attention Deficit Hyperactivity Disorder; ASD, Autism Spectrum Disorder; DCD; Developmental Coordination Disorder; CVI, Central Visual Impairment.

Outcome Measure	HIE (N=35)	Control (N=30)	P value
Cognitive Outcome			
WISC-V, full scale composite score, mean (SD)	100 (16.9)	112 (12.5)	0.005
Clinical Outcome			
Any neurological/neurodevelopmental diagnose and/or FSIQ < 85, No (%)	14 (40)	0 (0)	-
Cerebral Palsy, No (%)	3 (8.6)	0 (0)	-
FSIQ < 85, No (%)	6 (17.1)	0 (0)	-
ADHD, No (%)	4 (11.4)	0 (0)	-
ASD, No (%)	2 (5.7)	0 (0)	-
DCD, No (%)	4 (11.4)	0 (0)	-
Hearing impairment, No (%)	1 (2.9)	0 (0)	-
CVI, No (%)	1 (2.9)	0 (0)	-

Supplemental Table 3 Sensitivity analysis for surviving children without genetic syndrome exposed to hypothermia-treated neonatal hypoxic-ischemic encephalopathy (n=57) stratified by included or excluded from functional brain connectivity analysis.

Abbreviations: HIE, Hypoxic-Ischemic Encephalopathy; GA, Gestational Age; BW, Birth Weight; IQR, Intra Quartile Range; WISC, Wechsler Intelligence Scale in Children; IQ, Intelligence Quote.

Charectaristic	HIE included in analysis (n=35)	HIE excluded from analysis (n=22)	P value
GA, median (IQR), wk	40.6 (39.2 - 41.2)	40.5 (39.1 - 41.4)	0.75
BW, median (IQR), g	3500 (3326 - 3984)	3594 (3143 - 4340)	0.52
Apgar score at 10 minutes, median (IQR)	4 (2.5 - 6)	5 (4 - 6)	0.34
Sarnat grade of HIE			
Grade I, No (%)	0 (0)	4 (18.2)	
Grade II, No (%)	33 (94.3)	15 (68.2)	
Grade III, No (%)	2 (5.7)	3 (13.6)	0.015
Maximum respiratory support			
None, No (%)	8 (25.7)	4 (18.2)	
CPAP, No (%)	3 (8.8)	4 (18.2)	
IPPV, No (%)	16 (47.1)	12 (54.5)	0.47
HFO, No (%)	7 (20.6)	2 (9.1)	
Missing, No (%)	1 (2.9)	0 (0)	
Worst EEG background activity:			
Continuous, No (%)	8 (22.9)	1 (18.2)	
Discontinuous normal, No (%)	5 (14.3)	8 (36.4)	
Burst-suppression, No (%)	13 (37.1)	4 (18.2)	0.067
Low voltage, No (%)	2 (5.7)	3 (13.6)	
Flat trace, No (%)	3 (8.6)	2 (12.5)	
Missing, No (%)	4 (11.4)	4 (18.2)	
Neonatal seizures, No (%)	29 (82.9)	18 (81.8)	0.80[d]
EEG confirmed, No (%)	10 (54.3)	8 (36.4)	0.71[d]
Suspected (clinical), No (%)	19 (54.3)	10 (45.5)	
Any epileptic drug received, No (%)	32 (91.4)	20 (90.9)	0.68[d]
Phenobarbitone, No (%)	32 (91.4)	20 (90.9)	
Midazolam, No (%)	12 (34.3)	9 (40.9)	0.24
Lidocaine, No (%)	1 (2.9)	4 (18.2)	
Structural brain abnormality on MRI at age 10-12y, No (%)	24 (69%)	3 (60%)[a]	0.90[d]
Basal ganglia/Thalamus, No (%)	1 (3%)	0 (0%)[a]	-
Watershed, No (%)	14 (40%)	2 (40%)[a]	1.00
Solitary white matter lesions, No (%)	8 (23%)	0 (0%)[a]	-
Other, No (%)	1 (3%)	1 (20%)[a]	0.24
WISC IV, full scale IQ at 6 to 8 years, median (IQR)	102.5 (97 - 109)	107.5 (104 - 111)[b]	0.35
Any Neurologic/Neurodevelopmental diagnose and/or IQ < 85, No (%)	14 (40)	7 (31.8)[c]	0.61

[a]Available for 5 (23%) of the children
[b]Available for 12 (55%) of the children
[c]At age 6-8 years
[d]Yates correction applied in χ^2-test

Supplemental Figure 1. Flowchart of children included in the study with Sarnat grading of HIE severity in children exposed to Hypothermia-treated Hypoxic-Ischemic Encephalopathy (HIE).

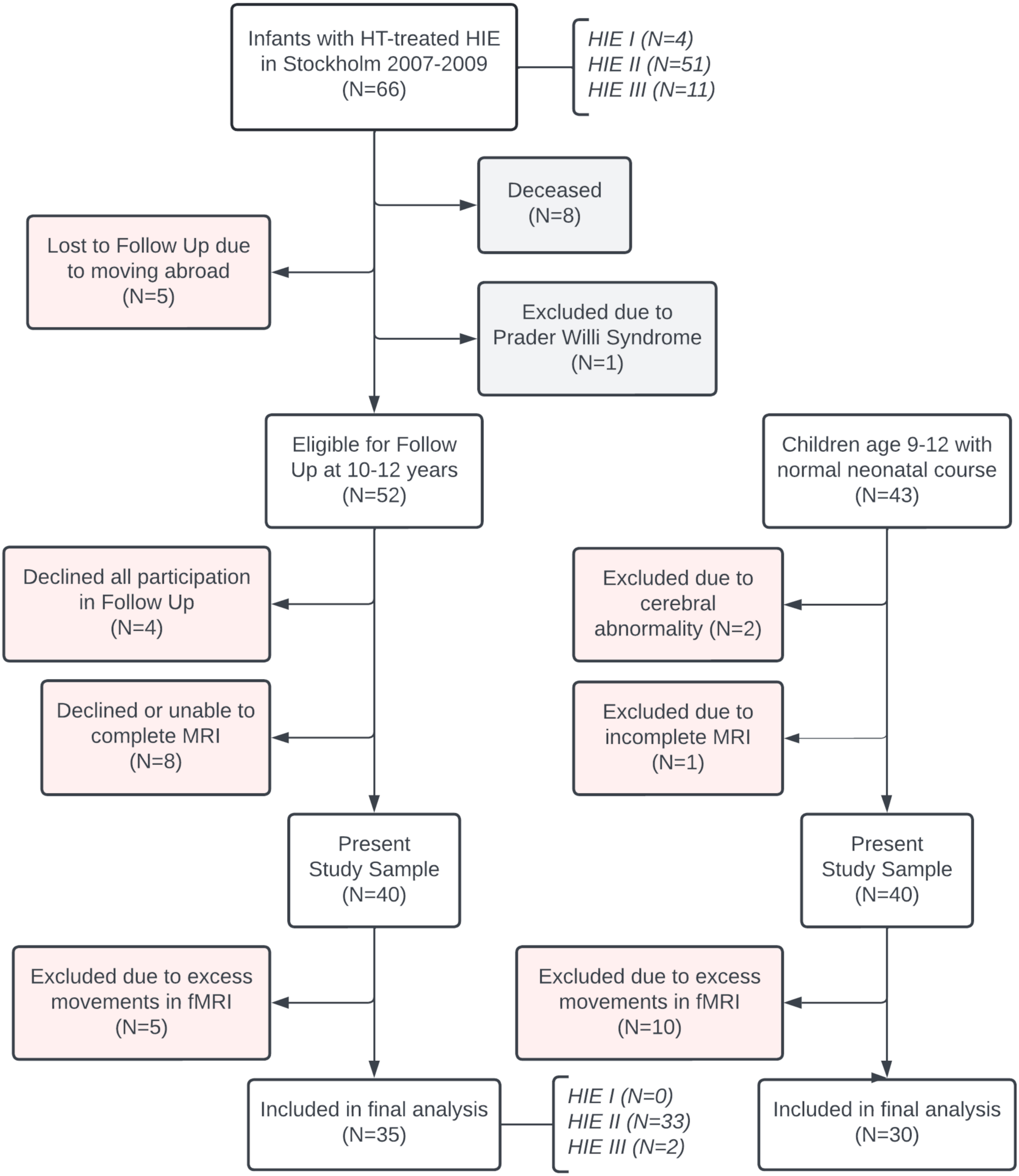

Supplemental Figure 2. All 15 resting-state functional connectivity networks from a canonical ICA analysis (with fixed dimensionality of 20 components) with the data from the HIE and control subjects combined into a single dataset.

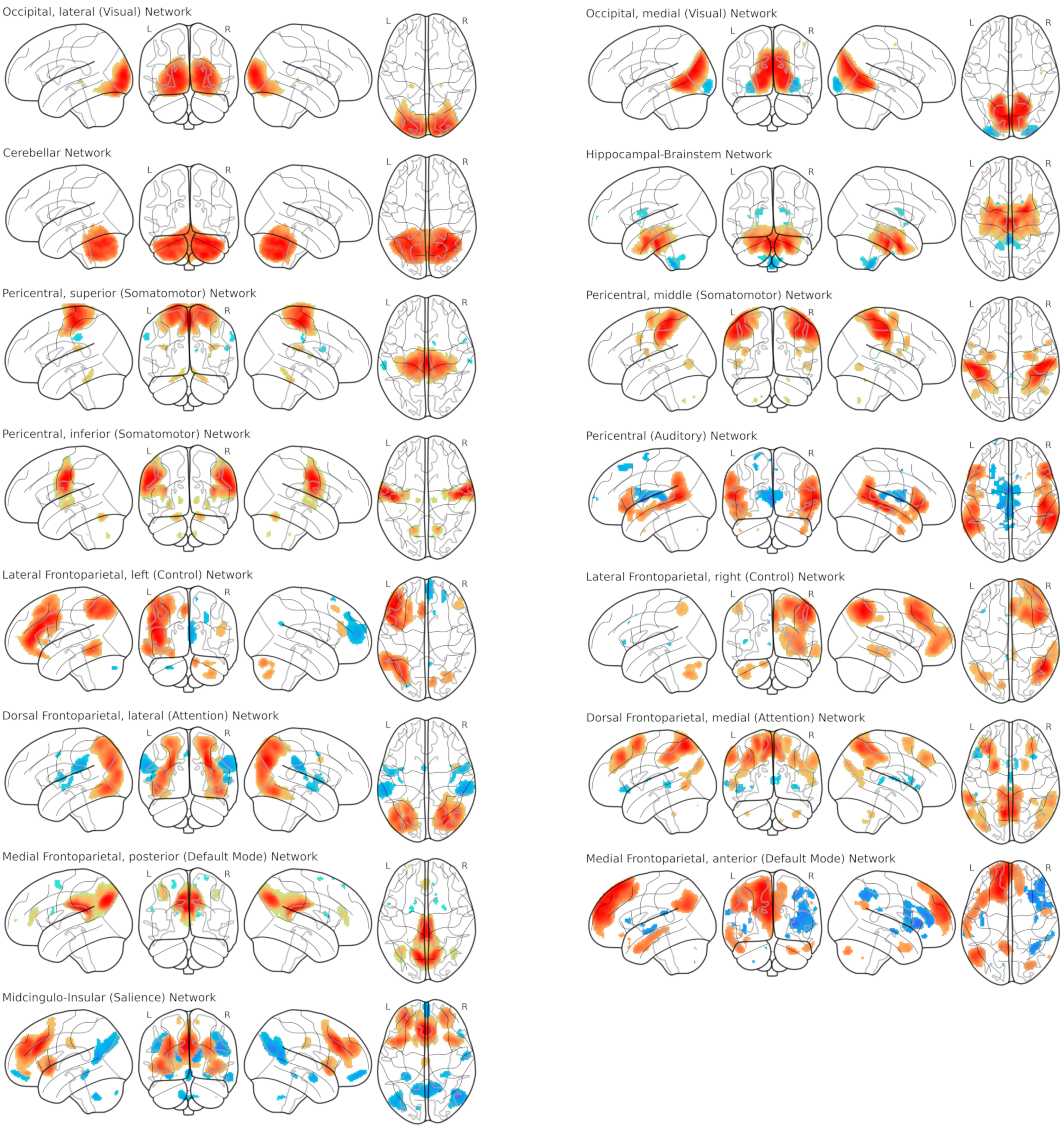

Supplemental Figure 3. Differences in functional connectivity between groups with and without exposure for Hypothermia-treated neonatal Hypoxic-Ischemic Encephalopathy before correcting for age. Results from non-parametric testing ($p<0.05$, voxel-wise threshold free cluster enhancement corrected (TFCE), minimal cluster size = 10) from data analysis with whole brain dual regression of the 15 ICA-derived networks (see Supplemental Figure 2) without covariates in the model detected clusters of decreased FC in children with hypothermia-treated HIE (N=35) compared to the control group (N=30) in four networks plotted on glass brain templates in A, with one cluster in the dorsolateral prefrontal cortex with decreased FC to the Medial Visual Network, plotted on anatomical template in B, surviving statistical correction for multiple comparisons across all networks (Bonferroni, 15 two-sided tests, $p<0.00167$). All significant changes are colored red, seed networks are colored blue in A, MNI coordinates are given in B.

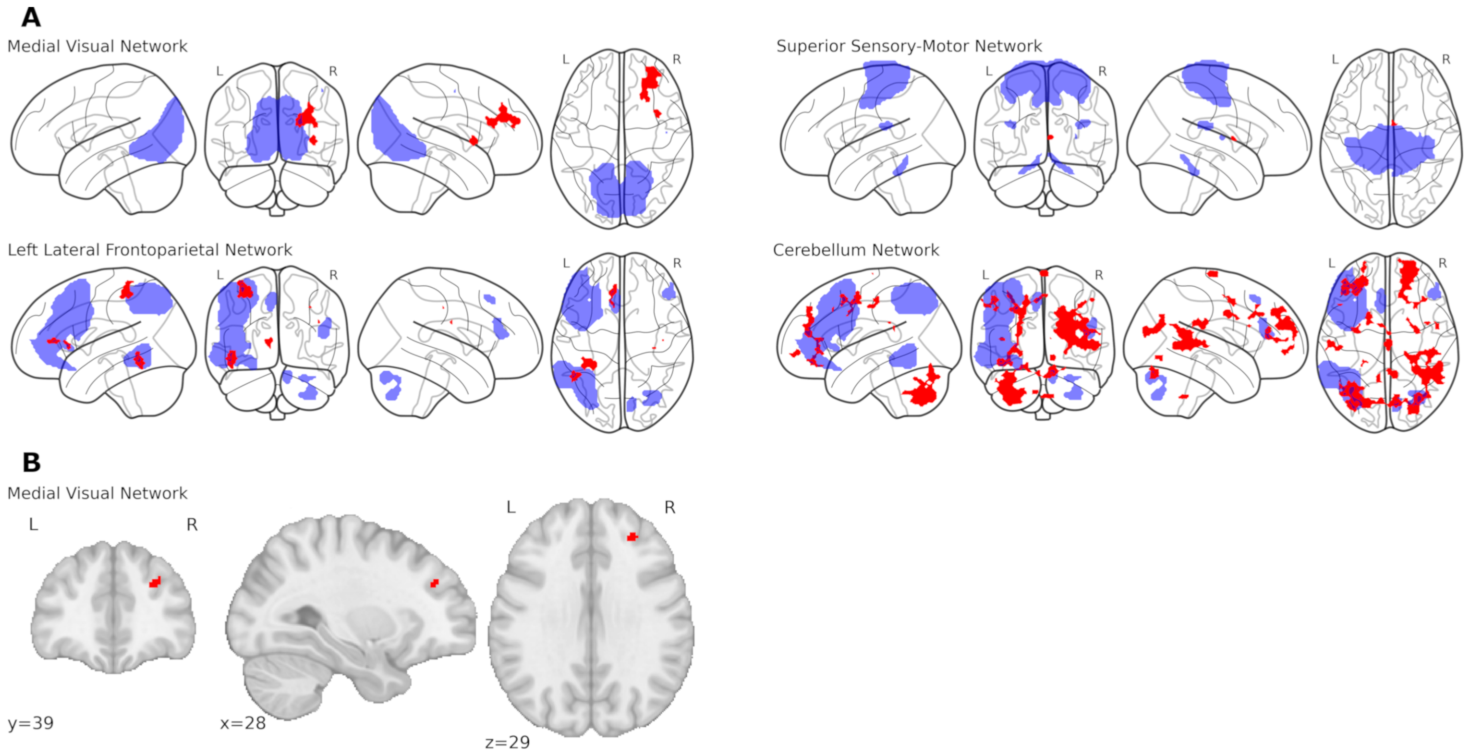

Supplement: Supplementary file 2 — Supplementary Tables 2, 3 and Figures [file 41390_2025_3951_MOESM2_ESM.pdf]
